# Epithelial expression of mRNA and protein for IL-6, IL-10 and TNF-α in endobronchial biopsies in horses with recurrent airway obstruction

**DOI:** 10.1186/1746-6148-4-8

**Published:** 2008-02-23

**Authors:** Miia Riihimäki, Amanda Raine, Jamshid Pourazar, Thomas Sandström, Tatiana Art, Pierre Lekeux, Laurent Couëtil, John Pringle

**Affiliations:** 1 Department of Clinical Sciences, Equine Internal Medicine, Swedish University of Agricultural Sciences, Uppsala, Sweden; 2 Dept. of Cell & Molecular Biology, Biomedical Centre, Uppsala, Sweden; 3 Department of Respiratory Medicine and Allergy, University Hospital, Umeå, Sweden; 4 Equine Sports Medicine Centre, Faculty of Veterinary Medicine, University of Liège, Belgium; 5 Department of Veterinary Clinical Sciences, School of Veterinary Medicine, Purdue University, West Lafayette, IN, USA; 6 Department of Clinical Sciences, Section of Large Animal Surgery and Medicine, Equine Internal Medicine, Swedish University of Agricultural Sciences, Box 7054, SE-750 07 Uppsala, Sweden

## Abstract

**Background:**

The aim of this study was to evaluate the contribution of bronchial epithelium to airway inflammation, with focus on mRNA and protein expression of cytokines of innate immunity IL-6, IL-10 and TNF-α, in horses with Recurrent Airway Obstruction (RAO) during exacerbation and in remission.

**Results:**

Despite marked clinical and physiologic alterations between exacerbation and after remission in the RAO horses no differences were detected in either cytokine mRNA or protein levels. Moreover, the expression of investigated cytokines in RAO horses on pasture did not differ from controls.

In comparing real-time PCR analysis to results of immunohistochemistry only IL-10 mRNA and protein levels in RAO horses on pasture were significantly correlated (r_s _= 0.893, p = 0.007). Curiously, in controls examined on pasture the TNF-α protein level was positively correlated to IL-10 mRNA expression (r_s _= 0.967, p = 0.007) and negatively correlated to IL-6 mRNA expression (r_s _= -0.971, p = 0.001).

**Conclusion:**

Given the complementary relationship of assessing cytokines directly by immunohistochemistry, or indirectly by PCR to mRNA, the lack of significant changes in either mRNA or protein levels of IL-6, IL-10 or TNF-α mRNA in RAO horses in exacerbation suggests that these particular cytokines in bronchial tissue may not play a substantive role in the active inflammation of this disease. To support this contention further studies examining time dependency of expression of IL-6, IL-10 or TNF-α are needed, as is expansion of the range of cytokines to include other key regulators of airway inflammation.

## Background

Bronchial epithelium acts not only as physical barrier but also is a key factor of remodelling and secretion of inflammation mediators in airways [1-4]. In addition to sampling methods such as bronchoalveolar lavage (BAL) and induced sputum, endobronchial biopsies have been used as a key research tool over the last decade to study the importance of bronchial epithelium in inflammatory diseases and define disease progression in asthma and COPD in humans [1,5-7], and evaluate the effects of different drug treatments and environmental effects on bronchial epithelium [8,9]. Besides identifying morphological changes, examination of bronchial tissue can provide information on mRNA expression and subsequent levels of translated inflammatory mediators directly within the tissue, both by resident cells and infiltrating inflammatory cells using immunohistochemistry (IHC) [10-12].

Horses are commonly affected by the disease "Recurrent Airway Obstruction (RAO)", which has many similarities with asthma in people. Inflammatory changes in the airways of horses with RAO have been studied predominately on the basis of cell samples collected by BAL, or less commonly, by analysis of other respiratory tract samples, such as bronchial brushing, tracheal lavage, exhaled breath condensate, and even lung tissue samples [13-19]. Most of our current understanding about the mechanism of inflammation and involvement of various regulatory or effector cytokines in the airways of horses with RAO has been derived from samples obtained by BAL. Apart from recent work of Ainsworth et al [14], similar assessment of bronchial tissues or direct identification of tissue cytokine levels by immunohistochemistry, which has been invaluable in human respiratory research, remains poorly investigated in horses.

The aim of this study was to evaluate the contribution of bronchial epithelium to airway inflammation in horses with RAO during exacerbation and in remission, with initial focus on relative cytokine mRNA and protein expression of cytokines IL-6, IL-10 and TNF-α, that are involved in innate non-specific immunity. The differences in mRNA levels measured by quantitative real-time PCR were compared with the corresponding protein levels in epithelial tissue measured by IHC. The epithelial cytokine levels in RAO horses during remission were also compared against samples from healthy controls on pasture.

## Results

### Clinical examination and pulmonary function test

There was no statistical difference in age and body weight in principal and control animals. The clinical score of RAO horses on pasture was statistically lower than during exacerbation (median ± SD, 7.00 ± 0.90 versus 3.00 ± 0.69, p = 0.02), but did not differ from control horses on pasture (3.00 ± 0.69 RAO versus 2.00 ± 0.00 pasture controls, p = 0.10). The RAO horses showed a significant worsening of pulmonary function during exacerbation as a response to provocation with mouldy hay, characterised by significant increase in ΔPpl_max _(41.80 ± 17.27 cmH_2_0 versus 9.70 ± 1.67 cmH_2_0, p = 0.02) and R_L _(2.96 ± 0.76 cmH_2_0/L/s versus 0.08 ± 0.02 cmH_2_0/L/s, p = 0.02). During respiratory exacerbation in RAO horses, there was also a significant decrease in C_dyn _after removing one outlier (0.21 ± 0.33 L/cmH_2_0, versus 1.53 ± 0.51 L/cmH_2_0 p = 0.036). During pasture the lung function improved in RAO horses, but remained significantly different when compared to healthy controls which had ΔPpl_max _of 5.89 ± 1.87 cmH_2_0, R_L _of 0.44 ± 0.12 cm H_2_0/L/s and C_dyn _of 1.54 ± 0.47 L/cmH_2_0.

### BAL cytology

The total cell count, percentage of neutrophils and total number of neutrophils in BAL was statistically higher (p = 0.02) in RAO horses post provocation compared to samples taken during remission on pasture (neutrophil percentage, 48.00 ± 13.31 versus 11.00 ± 10.66). During the pasture remission there was no statistical difference in BAL neutrophil percentage in RAO horses compared to controls (11.00 ± 10.66 versus 9.60 ± 6.82). Neither the percentage of neutrophils nor absolute number of neutrophils correlated with levels of investigated cytokine mRNA or protein expression. For the remaining cell types the percentage of macrophages showed a negative correlation with TNF-α mRNA expression in RAO horses during pasture (r_s _= -0.893, p = 0.007) but the biological relevance of this was of questionable significance.

### Immunohistochemistry of endobronchial biopsies

**Figure 1:**
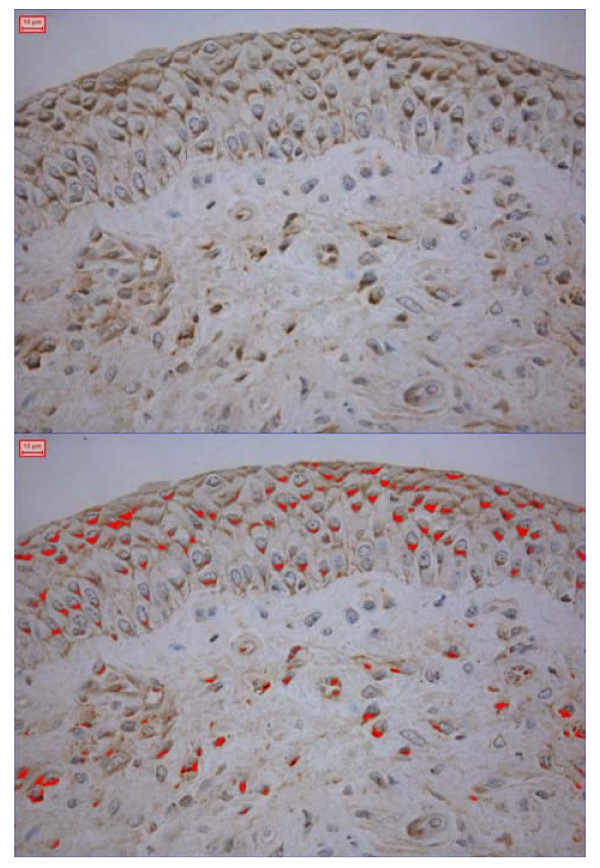
IL-10 expression in the bronchial epithelium of an RAO horse in exacerbation.

While there was a tendency for bronchial epithelial levels of IL-6 and TNF-α in RAO horses to increase during crisis the differences did not reach statistical significance (Table 1, Figure 1). Expression of investigated cytokines in RAO horses and controls on pasture did not differ, nor was any correlation found between IHC expression of different cytokines within individual horses.

**Table 1:** Bronchial epithelium expression of IL-6, IL-10 and TNF-α in RAO horses on crisis and in remission, and in control horses on pasture.

Cytokine	RAO provocation	RAO pasture	Controls pasture	RAO, p-value provocation/pasture
IL-6	1.08 (0.83–1.64)	0.63 (0.28–1.97)	0.94 (0.57–1.18)	p = 0.67, NS
IL-10	3.68 (2.46–6.11)	4.18 (2.28–5.38)	2.64 (1.77–5.95)	p = 0.21, NS
TNF-α	4.18 (1.26–5.03)	1.21 (0.95–2.51)	1.18 (0.54–2.10)	p = 0.45, NS

Immunohistochemical determination of percentage (%) of positively stained area of bronchial epithelium for selected cytokines. Values are given as medians (1st–3rd quartiles). NS = Not significant.

### Real-time PCR

**Figure 2:**
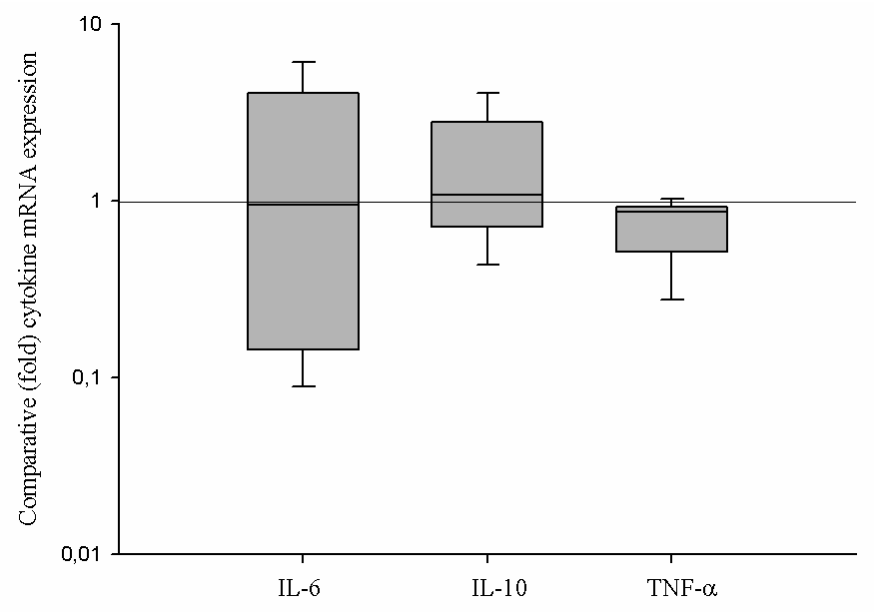
**Comparative (fold) cytokine mRNA expression**. The relative (fold) increase of cytokine mRNA levels (IL-6, IL-10 and TNF-α) is shown with interquartile range and line for median value. The horizontal line represents theoretical median value 1 (theoretically no difference between provocation and remission or ΔΔC_T _= 0, $2^{\Delta \Delta {\text{C}}_{\text{T}}}$ = 2^0 ^= 1).

The differences in cytokine mRNA levels from samples during crisis and on pasture are shown in Figure 2. In comparing RAO horses in crisis versus remission the only significant alteration in endobronchial tissue cytokine regulation was a 1.1 (median) fold decrease in TNF-α mRNA when in crisis (p = 0.035). The mRNA levels of investigated cytokines did not correlate with each other. Differences in expression of investigated cytokine mRNA in RAO horses and controls on pasture were not detected.

### Immunohistochemistry versus real-time PCR

IL-10 mRNA levels in RAO horses on pasture was significantly correlated with cytokine expression measured by IHC (r_s _= 0.893, p = 0.007) whereas IL-6 and TNF-α mRNA showed no correlation to their corresponding cytokines on IHC. Curiously, in controls examined on pasture, the TNF-α expression measured by IHC was positively correlated to IL-10 mRNA levels (r_s _= 0.967, p = 0.007) and negatively correlated to IL-6 mRNA levels (r_s _= -0.971, p = 0.001). In controls there was no correlation between investigated cytokines when comparing real-time PCR analysis to results of immunohistochemistry.

## Discussion

The horses in our study showed changes in pulmonary function and clinical scores that confirmed a period of respiratory crisis with neutrophilic inflammation and airway obstruction and also a period of remission. Surprisingly, we were unable to detect significant differences in either mRNA or protein expression in investigated cytokines in endobronchial tissue between provocation and remission. The only statistically significant finding was a small (1.1-fold down-regulation of TNF-α mRNA in endobronchial biopsies in RAO horses after provocation). However, given the extreme sensitivity of real time-PCR methodology this difference was unlikely of clinical relevance.

Earlier studies report conflicting results regarding IL-6 and IL-10 bioactivity or mRNA levels in BAL cells from RAO horses after challenge. However, these results may be due to differences in sampling time or the type of inhalation provocation [20,21]. Regarding IL-10, others have shown that inhalation challenge with lipopolysaccharide (LPS) elevates IL-10 mRNA expression in both BAL alveolar macrophages [20] and peripheral blood cells in RAO-susceptible horses [22]. However inhalation challenge with other agents failed to induce this up-regulation in BAL macrophages [20]. Thus it is possible that not only the sampling time, but also the mode and individual components of challenge agent may influence the dynamics of cytokine regulation. In our study, the negative correlation of bronchial TNF-α levels to IL-6 mRNA levels in controls examined on pasture may indicate the time-dependent changes in the expression of these two cytokines [23] or simply be coincidental. The positive correlation of IL-10 mRNA expression with TNF-α measured by IHC (r_s _= 0.967, p = 0.007) may have reflected the immunoregulatory properties of IL-10 as a counterbalance to TNF-α [24].

There are also several other possible reasons that this study did not detect alteration of these cytokines under exacerbation. Firstly, the time point for sample collection in correlation to provocation might have failed to capture a temporally related enhancement in examined cytokine expression. Others workers have shown time-dependent expression of inflammatory mediators in horse airways that occur within hours or days after provocation [13-16,18,20]. In contrast, our samples were collected only once RAO-horses had developed clinical signs of impaired pulmonary function, (ΔPpl_max _more than 1.5 kPa), rather than at a predetermined time after provocation. Alternatively, it can be questioned whether the bronchial epithelial tissues obtained were representative for the study. As shown in earlier studies in human COPD and asthma patients, there is variability of distribution of inflammatory cells in bronchial tissue which can require examination of biopsies from more than one airway generation to increase the statistical power to detect differences between individuals [25,26]. Of the six biopsies we obtained from each horse, the first two were from the first generation bronchus and examined by PCR. The remaining four biopsies were obtained progressively more distally in the lung and from these morphologically representative biopsies were then selected for further analysis by IHC. As well, the small biopsy size, the sampling of the epithelial layer without underlying deeper mucosa, and the fact that biopsies are generally sampled from more proximal airways and from subcarinae, are additional limitations to the sampling procedure. To secure antigen preservation and optimal morphology for IHC studies, we chose plastic resin embedding, GMA, used in several human studies together with acetone fixation containing protease inhibitors. This embedding technique ensures both excellent antigen preservation for IHC and morphologic overview [11]. When planning the study, the choice of equine antibodies was limited. At  present there are several equine specific antibodies available for the  investigated cytokines.

The primers used in PCR have been evaluated and validated in other studies. Moreover, companion studies in these RAO subjects have shown increased expression during crisis of other inflammatory mediators in BAL and epithelial cells collected by bronchial brushings and in biopsies [27-29], indicating ongoing inflammatory process. Thus, our findings suggest that, within the complex interrelation of regulatory cytokines, IL-6, IL-10 and TNF-α may not play a key role in the inflammation in bronchial tissues in horses with RAO.

Apart from IL-10 for the RAO horses at pasture, the lack of correlation of the cytokine mRNA to protein levels of IL-6, IL-10 and TNF-α was not unexpected as regulation of actual protein level is likely more complex than a direct relationship to amount of mRNA and the rate of translation and thus the amount of mRNA does not necessarily directly correlate with the level of protein expressed [30].

## Conclusion

These results show that combining quantitative real-time PCR and IHC methods on bronchial biopsy tissues can provide a new complementary research tool to study the role of mediators and structural elements in bronchial tissue associated with the inflammatory process in RAO-horses. Future work should focus on examining temporal events in the early stages following challenge exposure, and expanding the range of cytokines examined to study the role of airway epithelial cells in the immunopathogenesis of RAO.

## Methods

### Horses

Seven RAO horses with a history of recurrent episodes of respiratory distress when exposed to dusty stable environment (Mean weight 431 ± SD 90 kg, age 19.7 ± 6.6 years) and 6 healthy controls (Mean weight 475 ± SD 33 kg, age 16.8 ± 7.9 years) were included in the study. The RAO horses were sampled once clinical signs appeared following provocation with mouldy hay and straw bedding and secondly when in remission after two months on pasture, during which time six healthy horses were also similarly examined. RAO horses were sampled after provocation as previously described [31] when pulmonary function measurements indicated maximum change in transpulmonary pressure (ΔPpl_max_) above 1.5 kPa [32]. The clinical signs in horses were scored by a blinded observer according to an earlier reported system [32]. This study was approved by the institutional animal care and use committee, University of Liège, Belgium.

### Pulmonary function tests

Pleural pressure changes were measured with oesophageal balloon catheter connected to a calibrated pressure transducer and the air flow and volume measured through a facemask by a Fleish pneumotachograph (Model NR4; Gould Electronics, Eichstetten, Germany). The maximum transpulmonary pressure, pulmonary resistance and dynamic compliance were obtained from a lung function computer (Haemodynamics Respiratory System; ACEC, Namur, Belgium). Each measurement period averaged 2 min, and average values of 10 representative breaths were analysed.

### Bronchoalveolar lavage

After sedation with romifidine (0.01 mg/kg, iv, Sedivet; Boehringer Ingelheim, Ingelheim, Germany) and butorphanol tartrate (0.02 mg/kg IV, Torbugesic; Fort Dodge, Wyeth, Madison, New Jersey, USA) all horses had endoscopically (Pentax, Breda, The Netherlands, 170 cm × 12.9 mm) guided bronchial biopsy followed by BAL.

The BAL was performed with 6 × 60 ml sterile isotonic saline solution (37°C). Fluid recovered was placed on ice, samples pooled and aliquots collected for cytologic study. One aliquot was used to determine total leukocyte counts manually (THOMA cell; VWR International, Fontenay-sous-Bois, France). Another aliquot was centrifuged at 200 × g for 15 minutes (Shandon Cytospin Centrifuge, Thermo Electron Corporation, Pittsburgh, Pennsylvania, USA) and stained with May-Grünwald staining. Differential cell counts were determined by counting 200 cells per slide.

### Endobronchial biopsies

#### Sampling

Six biopsies (approximately 1–2 mm in diameter) were collected via endoscopy from the bronchial epithelium from subsegmental (3^rd ^to 4^th ^generation bronchi) and segmental subcarinae (1^st ^to 2^nd ^generation bronchi) airways with biopsy forceps (reusable, 240 cm, Pentax, KW3433S, Pentax Corporation, Tokyo, Japan). The first two biopsies from each horse were taken at the first generation bronchus and immediately frozen in liquid nitrogen and stored at -70°C until analysis for cytokine mRNA. The remaining biopsies were immediately placed in chilled acetone containing protease inhibitors for further preparation until immunostaining.

#### Biopsy Immunostaining

Biopsies were fixed in chilled acetone containing protease inhibitors (20 mM iodoacetamide and 2 mM phenyl methyl sulfonyl fluoride) at -20°C overnight. After fixation, biopsies were processed into glycolmethacrylate (GMA) resin [31] and stored at -20°C until cutting and immunostaining. Biopsies from all horses were processed with appropriate polyclonal  antibodies (Table 1) in the same batch of immunostaining. Two sections from one biopsy with optimal morphologic structure from each horse were cut at 2 μm thickness and placed on poly-L-Lysine treated slides. Endogenous peroxidises were inhibited using 0.1% sodium azide and 0.3% hydrogen peroxide for 30 minutes. After 3 × 5 minute washes in TRIS-buffered saline with 0.1% Triton-X-100 (TBST), non-specific antibody binding was blocked with undiluted culture medium, Dulbecco's Modified Eagle's Medium (Sigma, Missouri, US) containing 10% fetal calf serum and 1% bovine serum albumin (BSA) for 30 minutes. Further blocking of non-specific antibody binding was then done by incubation with swine normal serum for 30 minutes. The primary antibodies IL-6, IL-10 and TNF-α; (Table 2) diluted in 0.05% TBST with 1% BSA, were applied and incubated overnight. After rinsing with TBST for 3x5 minutes, biotinylated swine anti goat IgG F(ab´)2 (Dako Glostrup Denmark) was applied for 2 hours, followed by the streptavidin-biotin horseradish peroxidase complex (Dako) for another 2  hours. The sections were then visualized with diaminobenzidine (DAB) to yield a brown colour and counter-stained with Mayer Haematoxylin. TBST  treated biopsies were used as negative controls.

**Table 2:** Polyclonal antibodies used in IHC staining of bronchial epithelium.

**Anti-equine**	**Dilution**	**Source**
IL-6	1: 40	R & D SYSTEM, Abingdon, UK
IL-10	1: 25	R & D SYSTEM, Abingdon, UK
TNF-α	1: 40	R & D SYSTEM, Abingdon, UK

#### Quantification of epithelial expression of cytokines

Cytokine immunoreactivity was quantified using a colour video camera (Sony DXC-950P – Sony, Tokyo, Japan) containing 380,000 effective picture elements (pixels). The camera was connected to a Leica Imaging Workstation, with specific PC software (Leica Q500IW, Leica Cambridge UK). The image setting included a possibility to carefully adjust the individual colour components being displayed by the system in order to ensure a close match between the direct image and that being displayed. Detection of an appropriate colour was quantified using binary definition of colour images as displayed on the screen. The binary image required the user to define which pixel in the image that was to be considered for measurement. In short, the positively stained area was managed as a grid of pixels containing the binary value 1 (the pixel is "set") and remaining area contains the binary value 0 (the pixel is not "set"). All intact epithelium from both sections was used for quantification of epithelial area, from which positive immunoreactivity area was compared to the total measured area.

#### Quantitative real-time PCR

The biopsies for PCR were stored at -80°C until analysis. Isolation of total RNA from biopsies was performed with Trizol™ (Sigma-Aldrich Corp. AB, Stockholm, Sweden) according to the manufacturer's instructions. cDNA was synthesized from 500 ng total RNA using gene specific primers and Superscript III reverse transcriptase (Invitrogen)

Relative quantification of equine cytokine mRNA expression was performed by quantitative real-time PCR (RT-PCR) using a 7300 Real Time System (Applied Biosystem). Primer and probe sequences are listed in table 3. The RT-PCR reaction was run with Platinum Taq polymerase for 45 cycles of: 95°C, 15 s and 60°C, 1 min. All samples were run in triplicate and non template controls were included in each run. The RNA levels of the target genes were normalized against GAPDH RNA levels and the comparative C_T _($2^{-\Delta \Delta {\text{C}}_{\text{T}}}$) method was used for calculating relative cytokine mRNA expression. The PCR efficiencies, as determined by assaying serial dilutions of RNA, were approximately equal for the target genes and the housekeeping genes.

**Table 3:** Primer sequences and probes for real-time PCR assays.

***Gene***	***Forward primer 5' to 3'***	***Reverse primer 5' to 3'***	***Probe 5' to 3'***
**GAPDH**^a^	AAG TGG ATA TTG TCG CCA TCA AT	AAC TTG CCA TGG GTG GAA TC	TGA CCT CAA CTA CAT GGT CTA CAT GTT TCA
**IL-6**	AGC AAG TGT GAA AAC AGC AAG	CAT CAG GCA GGT CTC CTG AT	CTG GCA GAA AAC AAC CTG AAT CTT CCA
**IL-10**	TTC AGC AGG GTG AAG ACT TTC	CTT GGC AAC CCA GGT AAC CCT TA	TGT TGA ACG GGT CCC TGC TGG AG
**TNF-α**	GCT CCA GAC GGT GCT TGT G	GCC GAT CAC CCC AA AG TG	TGT CGC AGG AGC CAC CAC GCT

Primers and Probes designed from nucleotide sequence for this study. ^a^D.M. Ainsworth et al. Recurrent airway obstruction (RAO) in horses in characterised by IFN-γ and IL-8 production in bronchoalveolar lavage cells. In: Veterinary Immunology and Immunopathology 96 (2003) 83–91.

### Statistical analyses

Statistical comparisons were performed using a commercially available statistical software program (MINITAB). Because of the low number of animals data were treated nonparametrically. Descriptive statistics from each sampling period were calculated. The Wilcoxon signed rank test used to assess for differences between provocation and remission in RAO-horses, using a two tailed test (immunohistochemistry, BAL cytology, clinical score, pulmonary function), and descriptive data for the latter three categories presented as medians ± SD. The Wilcoxon signed rank test was also used for relative cytokine expression with PCR analysis where the theoretic median value 1 was used (theoretically no difference between provocation and remission or ΔΔC_T _= 0, $2^{\Delta \Delta {\text{C}}_{\text{T}}}$ = 2^0 ^= 1), where values over 1 indicate up regulation during provocation compared to pasture. The difference between mean ΔC_T _values for RAO-susceptible horses in remission and controls on pasture for each cytokine was used to count differences in cytokine mRNA expression and statistically tested using the Mann Whitney test. Spearman rank order correlation was used to evaluate the correlation in cytokine expression with PCR and IHC in biopsies and for correlation between cytokine expression and cytological findings. The results from BAL cell counts and pulmonary function from controls and principal animals during pasture were analysed using the Mann-Whitney test. A p-value < 0.05 was set as the level of significance.

## Authors' contributions

MR: Contributed to study design, clinical part of the study with sample collection and laboratory work, analyzed the data and drafted the manuscript.

AR: Performed the real-time PCR test and contributed to the data interpretation.

J. Pourazar: Performed the immunohistochemical analysis of endobronchial biopsies.

TA: Contributed to study design, coordination clinical part of the study and manuscript drafting.

TS, PL, LC: Contributed to study design, critical evaluation of data and manuscript drafting.

JP: Was involved in the study design and coordination, clinical part of the study with sample collection, critical evaluation of data and manuscript drafting.

All authors read and approved the final manuscript.
